# Comparative Analysis of Chemical Composition, Anti-Inflammatory Activity and Antitumor Activity in Essential Oils from *Siegesbeckia* *orientalis*, *S. glabrescens* and *S. pubescens* with an ITS Sequence Analysis

**DOI:** 10.3390/molecules23092185

**Published:** 2018-08-30

**Authors:** Xiaoxu Gao, Jiangchun Wei, Lina Hong, Sanpeng Fan, Gaosheng Hu, Jingming Jia

**Affiliations:** School of Traditional Chinese Materia Medica, Shenyang Pharmaceutical University, Shenyang 110016, China; 18842643805@163.com (X.G.); jiangchun_w@126.com (J.W.); honglina777@163.com (L.H.); threehedgehod@163.com (S.F.)

**Keywords:** *Siegesbeckia orientalis*, *S. glabrescens*, *S. pubescens*, essential oil composition, GC-MS, anti-inflammatory, antitumor, ITS1-5.8S-ITS2

## Abstract

Herba Siegesbeckiae (HS), derived from the aerial parts of three plants, *Siegesbeckia orientalis* (SO), *S. glabrescens* (SG), and *S. pubescens* (SP), has been used for the treatment of inflammatory diseases in China for centuries. In the present study, hydrodistillation was applied to extract essential oils from dried SO, SG, and SP aerial parts, and chemical composition analysis by gas chromatography–mass spectrometry (GC-MS) led to the identification of a total of 148 compounds (56 in SO, 62 in SG, and 59 in SP). The main components in the essential oils of SO, SG, and SP differed significantly. In vitro anti-inflammatory activity assays showed that SP essential oils (IC_50_, 0.97 μg/mL) significantly reduced the ability of lipopolysaccharide (LPS)-stimulated RAW264.7 macrophages to release NO, and the SO essential oil (IC_50_, 14.99 μg/mL) was better than the others at inhibiting the LPS-induced release of cytokine IL-6. Furthermore, the essential oils exhibited antitumor activities (IC_50_, 37.72–123.16 μg/mL) against Hep3B (liver) and Hela (cervical) cells. Linear regression analysis showed that, caryophyllene oxide peak area percentages showed remarkably high negative correlation coefficients with IC_50_ values of Hep3B and Hela cytotoxicity, which suggested the contribution of this compound on the cancer cell cytotoxicity of three essential oils. Finally, the ITS1-5.8S-ITS2 region was amplified and sequenced in order to generate genomic reference sequences for each plant. These can be used to identify the origins of the plants, and will assist other research studies related to these three plants.

## 1. Introduction

Plants produce a wide variety of volatile compounds, including terpenes, alcohols, aldehydes, esters, ethers, ketones, phenols, and oxides, that play important roles in defense, pollinator attraction, signal transduction, and so on. These volatile compounds are also important resources for the pharmaceutical, food, beverages, perfume, and cosmetics industries, and are thus economically significant [1,2,3]. The mixture of plant volatile compounds, which is also known as essential oil, can be extracted using hydrodistillation, steam distillation, supercritical fluid extraction, Soxhlet′s extraction, and ultrasound-assisted extraction [4,5].

Herba Siegesbeckiae (HS), a medicinal material used for the treatment of rheumatic arthritis, malaria, and snake bites in China since centuries ago [6], is derived from the aerial parts of three annual medicinal plants, *Siegesbeckia orientalis* (SO), *S. glabrescens* (SG), and *S. pubescens* (SP), which belong to the genus *Siegesbeckia* and the family Compositae. The main activities reported for HS extract are anti-inflammatory [7], antiallergic [8], antithrombotic [9], and immunosuppressive [10,11], but other activities have also been identified [12,13]. Recently, it was reported that SP essential oil significantly inhibited the proliferation of hepatocellular carcinoma cells (HepG2, Hep3B, Huh7, SMMC-7721). The 50% proliferation inhibition concentration of the SP essential oils was 42.0–95.2 μg/mL [14]. In the past few decades, through systematic chemical studies, three main categories of compound have been identified in HS: diterpenes, sesquiterpenes, and flavonoids [15,16,17,18]. Pharmacological studies suggested that diterpenoids are the main antirheumatic constituents of HS [19,20]. A series of ent-kaurane and ent-pimarane diterpenoids from HS has been reported [17,18,21,22,23]. However, to the best of our knowledge, a comparative study related to the chemical composition, anti-inflammatory activity, and antitumor activity of essential oils from the three individual plant species has not been reported.

In this study, the hydrodistillation method was used to extract essential oils from dried SO, SG, and SP aerial parts, and chemical composition analysis was carried out using gas chromatography–mass spectrometry (GC-MS). Subsequently, the bioactivities of the oils, including anti-inflammatory and antitumor activities, were evaluated in vitro. Furthermore, in order to provide a reference molecular marker for the plant origin, the ITS1-5.8S-ITS2 genomic regions of the three plants were amplified and sequenced. Our results revealed remarkable differences in the essential oil composition and bioactivities, and in the characteristic ITS sequence, which will facilitate the discrimination of the origins of the three plants and their subsequent utilization.

## 2. Results and Discussion

### 2.1. Analysis of Essential Oils

**Figure 1:**
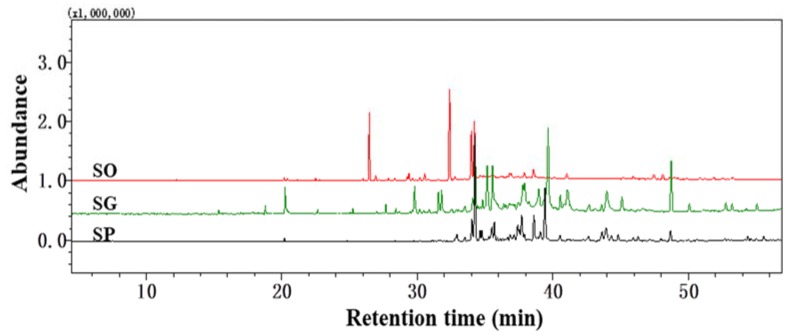
Representative GC-MS chromatograms of SO (red), SG (green), and SP (black) essential oils.

The yield of essential oils from the dried aerial tissue of the three plants was 0.06% (SO), 0.08% (SG), and 0.13% (SP) (*w*/*w*). Representative GC-MS chromatograms of the three essential oils are shown in Figure 1. A total of 56 (SO), 62 (SG), and 59 (SP) constituents were identified, representing 98.72% (SO), 93.89% (SG), and 91.35% (SP) of the total peak area. Across the three plants, 148 compounds were identified in total. The retention time, compound name, molecular formula, literature retention indices (*RL*), experimental retention indices relative to C_8_-C_20_ *n*-alkanes (*RI*), and peak area percentages are listed in Table 1.

As shown in Figure 1 and Table 1, the composition of the three essential oils differed significantly. The essential oil of SO was dominated by hydrocarbon sesquiterpenes (45.93%), oxygenated sesquiterpenes (44.99%), and oxygenated monoterpenes (1.53%), among which trans-α-bisabolene (24.41%), caryophyllene oxide (16.88%), caryophyllene (14.13%), and spathulenolspathulenol (12.46%) were found to be the main components. The SG essential oil mainly contained oxygenated sesquiterpenes (65.51%), hydrocarbon sesquiterpenes (9.79%), oxygenated diterpenes (9.26%), and oxygenated monoterpenes (4.90%), among which 4-(1,5-dimethylhex-4-enyl)cyclohex-2-enone (12.90%), cedrol (8.35%), phytone (7.83%), caryophyllene oxide (7.18%), dehydronerolidol (6.28%), and dehydrosaussurea lactone (5.49%) were the main components. The essential oil of SP was dominated by oxygenated sesquiterpenes (83.46%), oxygenated diterpenes (3.69%), and hydrocarbon sesquiterpenes (0.94%), with caryophyllene oxide (21.89%), germacra-4(15),5E,10(14)-trien-1β-ol (14.10%), trans-longipinocarveol (5.87%), (−)-spathulenol (5.14%), and dehydrosaussurea lactone (4.85%) as the main components.

**Figure 2:**
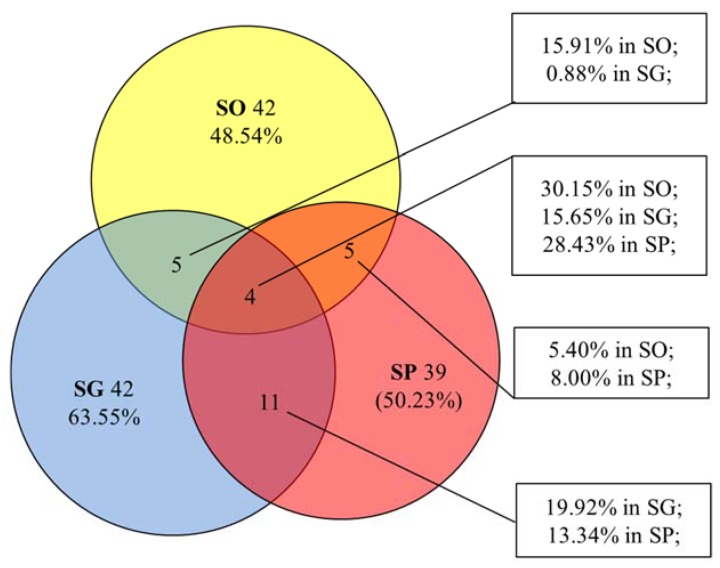
Venn diagram of essential oil compositions of SO, SG, and SP.

Only four chemical components were in common among all three species, namely nerol, spathulenol, caryophyllene oxide, and humulene epoxide. These accounted for 30.15%, 15.65%, and 28.43% of the total peak area in SO, SG, and SP essential oils, respectively (Figure 2). The essential oils of SO and SG shared 5 chemical components, as did the essential oils of SO and SP (Figure 2). SG and SP shared eleven identical components, as shown in Figure 2.

### 2.2. Anti-Inflammatory Activity

According to traditional use and recently published results, anti-inflammatory [7], antiallergic [8], antithrombotic [9], and immunosuppressive [10,11] effects are reported as the main activities of HS. Diterpenoids were the main antirheumatic constituents of HS [19], of which kirenol was the main chemical constituent. Pharmacological studies suggested that kirenol could inhibit paw swelling, boost up the proliferation of splenic lymphocyte of adjuvant arthritis rats, regulate T lymphocyte subpopulation ratio, and inhibit specific immune response of normal mice [20]. As we know, NO plays an important role in the inflammatory, immune responses, and in thrombotic pathogenesis processes. Inhibition of excess NO production has been used as an assay in the screening of anti-inflammatory, antithrombotic, and immunosuppressive drugs. Therefore, in this study, we treated the mouse macrophage cell line RAW264.7 with lipopolysaccharide (LPS) to induce an inflammatory response and we investigated the inhibitory effects of the three essential oils on NO production by the activated cells. Before the anti-inflammatory activity assay, the cytotoxicity of the three essential oils was evaluated by MTT assay. Our results suggested that the three essential oils had no obvious cytotoxic effects on the RAW264.7 cell line when the concentration was lower than 200 μg/mL (shown in Figure S1). 

**Figure 3:**
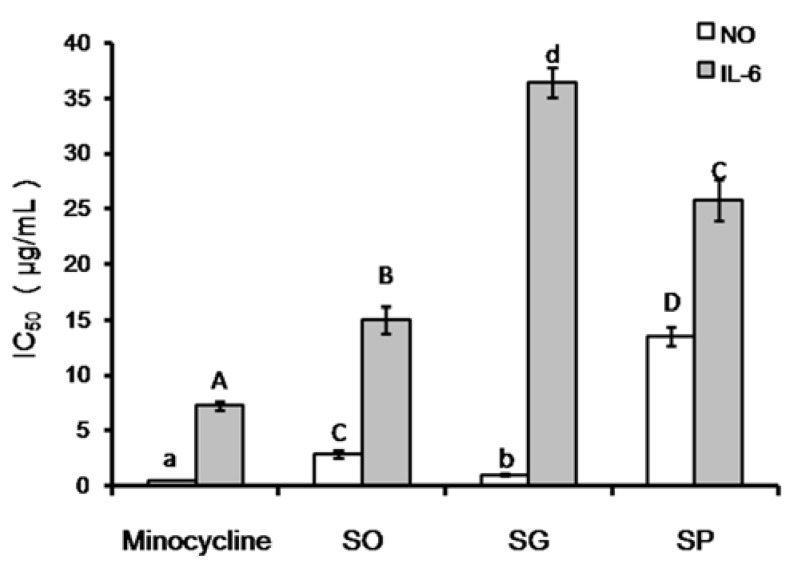
Anti-inflammatory effect of the SO, SG, and SP essential oils. A–D, *p* < 0.01; a,b,d, *p* < 0.05.

As shown in Figure 3, the positive control minocycline showed the strongest NO inhibition activity with IC_50_ = 0.37 μg/mL, which is significantly lower than SG oil (*p* < 0.05), SO oil (*p* < 0.01), and SP oil (*p* < 0.01). Among the three essential oils, SG oil showed the lowest IC_50_ of 0.97 μg/mL, which is significantly lower (*p* < 0.01) than SO oil (IC_50_ = 2.83 μg/mL) and SP oil (IC_50_ = 13.48 μg/mL). Even though the SG oil showed weaker NO inhibition activity than minocycline, it still has the strongest NO inhibition effect of any crude essential oil as far as we know.

Inflammatory cells can release a variety of inflammatory cytokines which participate in the regulation of the body’s innate immune response and directly kill target cells or mediate apoptosis, thus promoting the repair of damaged tissues. One of these cytokines is IL-6, a critical component of the inflammatory mediator network which plays an important role in the inflammatory response. The ability of the essential oils to inhibit the release of IL-6 was tested in LPS-treated RAW264.7 cells. As indicated in Figure 3, the ability of SO essential oil (IC_50_, 14.99 μg/mL) to inhibit the release of cytokine IL-6 was significantly weaker (*p* < 0.01) than that of the positive control (minocycline) (IC_50_, 7.21 μg/mL), but stronger (*p* < 0.01) than that of SP (IC_50_, 25.76 μg/mL) and SG (IC_50_, 36.41 μg/mL) essential oils.

NO and cytokine IL-6 are important indicators of the cellular inflammatory response. Our results demonstrated that the three essential oils showed significant anti-inflammatory activity. In summary, the three essential oils significantly inhibited the LPS-induced secretion of NO and cytokine IL-6 by the macrophage cell line RAW264.7. These results provide important clues for the identification of possible candidate compounds from the three essential oils, especially SG and SO essential oils.

### 2.3. Antitumor Activity 

**Figure 4:**
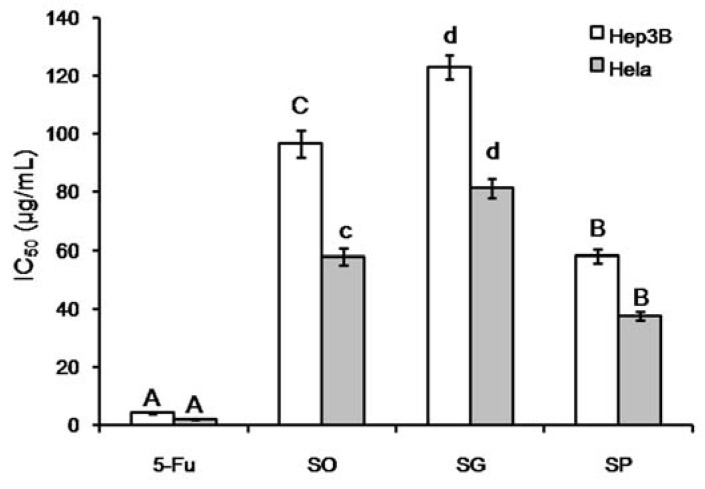
Cytotoxicity of the essential oils of SO, SG, and SP against Hep3B and Hela cell lines. A–C, *p* < 0.01; c,d, *p* < 0.05.

The in vitro antitumor activities of the essential oils were evaluated using two cancer cell lines, Hep3B (liver) and Hela (cervical). The cytotoxicity was expressed as IC_50_ values, as shown in Figure 4. The IC_50_ of the positive control 5-Fu (4.19 μg/mL in Hep3B, 2.08 μg/mL in Hela) was significantly lower than the three essential oils tested (*p* < 0.01). Among the three essential oils, SP exhibited the strongest cytotoxicity (38.10 μg/mL for Hep3B, 37.72 μg/mL for Hela). These results suggest that essential oils from the three plants might be used as a natural source of compounds for anticancer compound screening. 

A recent study [24] showed that SO ethanol extract significantly inhibited the proliferation of *RL* 95-2 human endometrial cancer cells. Furthermore, SO ethanol extract was effective against A549 (lung cancer), HepG2 (hepatpma), MDA-MB-231 (breast cancer), and especially on LNCaP (prostate cancer) cell lines. At the same time, it was reported that caryophyllene oxide (IC_50_ value = 14.90 μg/mL) and caryophyllene (IC_50_ value = 33.20 μg/mL) were mainly responsible for most cytotoxic activity of SO ethanol extract against *RL* 95-2 cells. As mentioned previously in Section 2.1, there are only four common compounds in the three essential oils, and caryophyllene oxide is one of the four common components in the three essential oils. 

In order to find out the possible relationships between common compounds and two kinds of activities, a linear regression analysis was carried out between percentages and IC_50_ values. Our results showed that (Figure S2) only caryophyllene oxide peak area percentages showed remarkably strong negative correlation coefficients with IC_50_ of NO inhibition (R^2^ = 0.959), Hep3B (R^2^ = 0.918), and Hela (R^2^ = 0.980) cytotoxicity. These results suggested the possible important contribution of caryophyllene oxide on the anticancer activity and anti-inflammatory effects of Herba Siegesbeckiae essential oil. 

### 2.4. ITS1-5.8S-ITS2 Sequence

Due to the similarity in morphological characteristics of dried and chopped commercial materials, it is difficult to distinguish the three plants from each other. Therefore, it is important to provide a reliable molecular marker for the identification of the plant origin and for comparison with other research studies. It has been accepted that genomic ribosomal DNA sequences, especially ITS1-5.8S-ITS2, can be used for the interspecific identification of plant origin. Therefore, the full ITS1-5.8S-ITS2 sequence was amplified from genomic DNA of the three plants and sequenced. The accession numbers in Genbank are MH701787 (SO), MH701847 (SG), and MH701848 (SP). Multiple alignment of the ITS1-5.8S-ITS2 sequence from the three plants was carried out using DNAMAN 9.0 software. As shown in Figure S3, across the total sequence of 730–732 bp, the three plants shared 99.27% similarity with each other. There are 16 single nucleotide polyphorism (SNP), which can be used for the development of specific Polymerase chain reaction (PCR) assays to distinguish the origins of samples of these three plants.

## 3. Conclusions

In this study, hydrodistillation was used to extract essential oils from the dried aerial parts of SO, SG, and SP, and their chemical compositions and bioactivities were analyzed and compared. A total of 56, 62, and 59 constituents were identified in SO, SG, and SP essential oils, respectively, by GC-MS. There was a marked difference between the components of the three essential oils. The essential oils of SO, SG, and SP exhibited various levels of anti-inflammatory and antitumor activity in vitro. The essential oil of SP (IC_50_, 0.97 μg/mL) significantly reduced NO release by LPS-induced RAW264.7 macrophages, and the essential oil of SO was the most effective at reducing the release of cytokine IL-6. The antitumor activities of the essential oil from SP were much better than SG and SO. Therefore, the essential oils of SO, SG, and SP were significantly different from each other in terms of phytochemical composition, but with similar bioactivities with various activity levels. Among four common compounds in three essential oils, caryophyllene oxide peak area percentages showed remarkably high negative correlation coefficients with IC_50_ values of Hep3B and Hela cytotoxicity, which suggested the contribution of this compound on the cancer cell cytotoxicity of three essential oils. Finally, in order to provide detailed information about the origin of the plants used in the present work, the complete ITS1-5.8S-ITS2 region was amplified from plant genomic DNA and sequenced. This sequence can be used by other researchers to confirm whether their plant specimens have the same origin as ours or not.

## 4. Materials and Methods 

### 4.1. Materials

The flowering SP and SG plant materials were collected in October 2017 from Jinzhai, Anhui province, China. The flowering SO plant material was collected in April 2018 from Zhanzhou, Hainan province, China. Voucher specimens were deposited at the Herbarium of Shenyang Pharmaceutical University (Shenyang, China). The voucher numbers were SYPU-P-201710-007 (SP), SYPU-P-201710-008 (SG), and SYPU-P-201804-025 (SO). They were identified by Associate Professor Jia Lingyun in Shenyang Pharmaceutical University (Shenyang, China). The fresh plants were air-dried and ground into a fine powder which was passed through a 20 mesh and stored in sealed plastic bags at −20 °C for future use. RAW264.7, a mouse macrophage cell line, was purchased from the Cell Bank of the Shanghai Institute of Cell Biology and Biochemistry, Chinese Academy of Sciences (Shanghai, China). The cancer cells lines Hep3B (liver) and Hela (cervical) were obtained from the Cell Bank of the Chinese Academy of Sciences (Shanghai, China).

### 4.2. Reagents

He (purity > 99.999%) and N_2_ (purity > 99.999%) were supplied by Shenyang Qianzhen Chemical Gas (Shenyang, China). Hexane (HPLC grade) and anhydrous sodium sulfate (analytical grade) were purchased from Shandong Yuwang Chemical Group (Shandong, China). Plant Genomic DNA Extraction Kits, 2X Taq master premix, and GoldView dye were purchased from Kangweishiji Bio. (Beijing, China). Agarose G-10 powder was purchased from Biowest (Hongkong, China). Synthesis of primers ITS1 and ITS4 as well as Sanger sequencing were carried out at Genewiz Bio. Ltd. (Suzhou, China). DMSO and 1% penicillin-streptomycin antibiotics were purchased from Sigma (biology grade, Burlington, VT, USA). RPMI 1640, Eagle’s Minimum Essential Medium, and 10% fetal bovine serum were purchased from Gibco (Waltham, MA, USA). Nitric Oxide Assay Kit was purchased from Beyotime Institute of Biotechnology (Jiangsu, China). ELISA kits were purchased from Shanghai Joyee Biotechnics Co., Ltd. (Shanghai, China). Minocycline was purchased from Dalian Meilun Biotech Co., Ltd. (Dalian, China). 5-Fluorouracil was purchased from Shanghai Yuanye Bio-Technology Co., Ltd. (Shanghai, China).

### 4.3. DNA Extraction, ITS Amplification, Electrophoresis and Sequencing

Dried leaf tissues were ground into a fine powder. Then, DNA was extracted using a Plant Genomic DNA Extraction Kit following the manufacturer′s instructions. Extracted DNA (1 μL) was used as the template for PCR amplification, with 25 μL 2X Taq master premix, 3 μL 10 μM ITS1 primer (5′-TCCGTAGGTGAACCTGCGG-3′), 3 μL 10 μM ITS4 primer (5′-TCCTCCGCTT ATTGATATGC-3′), and 18 μL double-distilled H_2_O. The amplification conditions were as follows: 94 °C for 5 min (1 cycle); 94 °C for 30 s, 55 °C for 30 s, and 72 °C for 40 s (30 cycles); 72 °C for 10 min (1 cycle). PCR product (5 μL) was applied to a 1% agarose gel containing GoldView dye and electrophoresis was carried out for 15 min at 150 V followed by observation under UV light in a Tenon gel imaging system. After confirmation of a strong, single amplified band of about 700 bp, the PCR product was sent for Sanger sequencing using ITS1 and ITS4 as sequencing primers. Sequences were confirmed by comparing them with the original sequencing chromatogram and were connected using Seqman software (Madison, MI, USA).

### 4.4. Essential Oil Extraction

100 g powder of dried aerial parts were added together with 1 L distilled water into a 2 L round-bottomed bottle, which was connected to a Clevenger-type apparatus with tap water for cooling. The hydrodistillation was continued for 4 h to extract the essential oils. The obtained essential oil was collected from the side arm and dried with anhydrous sodium sulphate, then sealed in a headspace bottle and stored at 4 °C until it was tested.

### 4.5. Gas Chromatography-Mass Spectrometry (GC-MS) Analysis

The GC-MS analysis was carried out on a Shimadzu (TQ-8040) series GC-MS system (Tokyo, Japan) equipped with an AOC-20i autosampler. The columns used an HP-5 capillary column (30 m × 0.32 mm, i.d. 0.25 μm) with a stationary phase of 5% diphenyl/95% dimethylpolysiloxane. Helium was used as the carrier gas. The column temperature was maintained at 40 °C for 3 min and then programmed to increase as follows: rising from 40 °C to 130 °C at a rate of 4 °C/min and holding for 2 min; rising from 130 °C to 150 °C at a rate of 2 °C/min and holding for 3 min; rising from 150 °C to 180 °C at a rate of 2 °C/min and holding for 3 min; and finally rising from 180 °C to 210 °C at a rate of 5 °C/min and holding for 5 min. The ionization energy was 70 eV with a scan time of 0.3 s and a mass range of 45–500 AMU. The identification of the compounds was based on the comparison of their retention times and mass spectra with those from the NIST14 and NIST14s (National Institute of Standards and Technologies, Mass Spectra Libraries, Gaithersburg, MD, USA) [25].

### 4.6. Anti-Inflammatory Activities

#### 4.6.1. Cell Line and Cell Culture

RAW264.7, a mouse macrophage cell line, was grown in RPMI 1640 supplemented with 10% heat-inactivated fetal bovine serum, 100 U/mL penicillin, and 100 µg/mL streptomycin in a 37 °C humidified incubator containing 5% CO_2_.

#### 4.6.2. Cell Viability Assay

The MTT assay was used to evaluate the effect of essential oils on cell viability [26]. RAW264.7 cells were seeded in 96-well plates at a density of 5 × 10^4^ cells/well. After overnight growth, cells were treated with different concentrations of essential oils for 1 h, then incubated in the presence or absence of LPS (100 ng/mL) for the next 24 h. Then, 20 μL of MTT solution (5 mg/mL) was added and the cells were cultured for a further 4 h. After that, the supernatant was carefully removed and then the resulting formed azan crystals were dissolved in 100 µL DMSO with horizontal shaking. The absorbance at 490 nm (ref. 570 nm) [24] was measured with a microplate reader.

#### 4.6.3. Measurement of NO and IL-6 Release

RAW264.7 cells were seeded into 96-well plates at a density of 8 × 10^4^ cells/mL and cultured overnight. After pretreatment with different concentrations of essential oils (0.2–200 µg/mL) or the positive control (minocycline) for 1 h, the cells were stimulated with LPS (100 ng/mL) for 24 h. The concentration of NO in the conditioned culture medium was examined with the Nitric Oxide Assay Kit and the release of IL-6 in the supernatants was assayed using ELISA kits according to the manufacturer’s instructions [27,28]. The concentrations were calculated from standard curves.

### 4.7. Antitumor Assay

#### 4.7.1. Cell Lines and Cell Culture

The cancer cells lines Hep3B (liver) and Hela (cervical) were grown in media supplemented with 10% fetal bovine serum and 1% penicillin-streptomycin antibiotics and maintained in a CO_2_ incubator at 37 °C in 5% CO_2_. The medium was changed every 2 days until the cells reached confluence, at which point they were subcultured.

#### 4.7.2. Cytotoxicity Assay 

The essential oils from HS were evaluated for their cytotoxic activities against cells using the MTT cell viability assay with 5-fluorouracil as the positive control. First, cellular suspensions (1 × 10^5^ cells/mL) were cultured in 96-well plates and exposed to different concentrations of the essential oils (1–200 μg/mL) using 3 wells for each concentration. The plates were incubated at 37 °C in 5% CO_2_ for 48 h [29]. Then, 200 μL MTT solution was added to each well and the plates were cultured for 4 h. The absorbance of each well was measured at 490 nm using a microplate reader. The data were expressed as the mean percentage of viable cells as compared to the respective control cultures treated with the solvent. The half maximal growth inhibitory concentration (IC_50_ values) was calculated from the standard curves of the dose-dependent cytotoxicity of the essential oils.

### 4.8. Statistical Analysis

All activity assay experiments were repeated three times and the data were represented as mean ± SD. Significance analysis was carried out using the t-test in Microsoft Excel 2007. Letters above the columns in Figure 2 and Figure 3 represent significance (A–D: *p* < 0.01; a,b,d: *p* < 0.05)

## Figures and Tables

**Table 1:** Chemical composition (peak area percentage) of the essential oils of SO, SG, and SP.

No.	*Rt* (min)	Compound Name	Molecular Formula	*RL*	*RI*	SO	SG	SP
1	6.14	(3E)-Hexenol	C_6_H_12_O	855	868		0.05	
2	6.59	Hexanol	C_6_H_14_O	868	860		0.04	
3	6.95	Dibutyl oxide	C_8_H_18_O	878	882			0.09
4	7.49	Butyl acrylate	C_7_H_12_O_2_	893	884			0.11
5	7.96	Butyl propionate	C_7_H_14_O_2_	906	894			0.02
6	11.01	Nopinene	C_10_H_16_	992	978			0.03
7	11.28	Butyl butyrate	C_8_H_16_O_2_	1000	984			0.07
8	12.24	α-Cymene	C_10_H_14_	1025	1027	0.21		
9	12.41	Clearene	C_10_H_16_	1029	1018			0.07
10	13.57	6-Ethyl-2-methyloctane	C_11_H_24_	1059	1046			0.03
11	13.61	2,4-Dimethyldecane	C_12_H_26_	1060	1086		0.03	
12	15.35	Linalool	C_10_H_18_O	1105	1082		0.33	
13	15.58	Dehydrocineole	C_10_H_16_O	1111	1041		0.03	
14	16.45	4-Acetyl-1-methylcyclohexene	C_9_H_14_O	1133	1118	0.12		
15	16.96	Camphor	C_10_H_16_O	1147	1121		0.07	
16	17.20	Mentha-1,5-diene-8-ol	C_10_H_16_O	1153	1125		0.04	
17	17.39	Neryl oxide	C_10_H_16_O	1158	1135	0.11		
18	17.78	2-Camphanol	C_10_H_18_O	1168	1165			0.04
19	17.83	Borneol	C_10_H_18_O	1169	1165		0.16	
20	17.90	β-Phellandren-8-ol	C_10_H_16_O	1171	1178		0.13	
**21**	**18.20**	**4-terpineol**	**C_10_H_18_O**	**1179**	**1174**	**0.01**	**0.07**	
22	18.55	p-Cymen-8-ol	C_10_H_14_O	1188	1197	0.07		
23	18.73	(−)-α-Terpineol	C_10_H_18_O	1192	1196	0.08		
**24**	**18.78**	**α-terpineol**	**C_10_H_18_O**	**1194**	**1194**		**0.71**	**0.04**
25	18.83	2-Decanone	C_10_H_20_O	1195	1181	0.09		
26	19.16	2-Decanol	C_10_H_22_O	1204	1218	0.11		
**27**	**20.18**	**Nerol**	**C_10_H_18_O**	**1233**	**1228**	**0.56**	**2.85**	**0.48**
28	20.42	O-Methylthymol	C_11_H_16_O	1239	1231	0.42		
29	20.97	2-Hexanoylfuran	C_10_H_14_O_2_	1255	1265			0.04
**30**	**21.16**	**Geraniol**	**C_10_H_18_O**	**1260**	**1256**	**0.27**		**0.07**
31	21.72	2,4-Dimethylphenethyl alcohol	C_10_H_14_O	1276	1292	0.01		
32	21.76	Dihydro-4-(3-methyl-2-methylenebutyl)-2(3*H*)-furanone	C_10_H_16_O	1277	1286		0.02	
33	21.98	4-Methyldodecane	C_13_H_28_	1284	1269			0.03
34	22.02	4,6-Dimethyldodecane	C_13_H_30_	1285	1285		0.04	
35	22.49	2-Undecanone	C_11_H_22_O	1298	1295	0.58		
36	22.65	Thymol	C_10_H_14_O	1302	1293		0.33	
37	22.77	2-Undecanol	C_11_H_24_O	1306	1297	0.23		
38	23.33	(E,E)-2,4-Decadienal	C_10_H_16_O	1322	1321		0.03	
39	24.71	Hexahydropseudoionone	C_13_H_26_O	1361	1341	0.05		
**40**	**24.85**	**Eugenol**	**C_10_H_12_O_2_**	**1365**	**1359**		**0.03**	**0.11**
41	25.05	Nerol acetate	C_12_H_20_O_2_	1370	1352		0.13	
42	25.24	α-Cubebene	C_15_H_24_	1376	1355		0.42	
43	25.98	2-Dodecanone	C_12_H_24_O	1397	1396	0.45		
44	26.48	4,8,8-Trimethyl-2-methylene-4-vinylbicyclo[5.2.0]nonane	C_15_H_24_	1409	1407			0.10
45	26.93	cis-Caryophyllene	C_15_H_24_	1419	1425	1.30		
**46**	**27.00**	**Caryophyllene**	**C_15_H_24_**	**1420**	**1418**	**14.13**	**0.21**	
**47**	**27.19**	**1,4-Dimethoxy-2-tert-butylbenzene**	**C_12_H_18_O_2_**	**1425**	**1406**	**0.21**	**0.02**	
48	27.67	trans-α-Bergamotene	C_15_H_24_	1436	1430		0.89	
49	27.72	Aromandendrene	C_15_H_24_	1437	1416	0.23		
50	27.88	γ-Elemene	C_15_H_24_	1440	1431	0.62		
51	28.34	cis-α-Bisabolene	C_15_H_24_	1451	1478	0.56		
52	28.37	α-Farnesene	C_15_H_24_	1451	1458			0.12
53	28.56	(E)-β-Famesene	C_15_H_24_	1456	1440		0.13	
54	29.26	Acoradiene	C_15_H_24_	1471	1474	0.61		
55	29.39	γ-Muurolene	C_15_H_24_	1474	1483	1.42	0.28	
56	29.45	Guaia-1(10),11-diene	C_15_H_24_	1476	1490		0.21	
57	29.57	1,5-Cadinadiene	C_15_H_24_	1479	1460			0.11
58	29.64	Curcumene	C_15_H_22_	1480	1524	0.51		
59	29.8	cis-β-Farnesene	C_15_H_24_	1484	1458		3.96	
60	30.15	γ-Curcumene	C_15_H_24_	1492	1486		0.53	
61	30.18	β-Funebrene	C_15_H_24_	1492	1479			0.15
62	30.19	Elemol	C_15_H_26_O	1492	1512	0.72		
63	30.47	β-Himachalene	C_15_H_24_	1499	1518		0.19	
64	30.54	α-Bisabolene	C_15_H_24_	1500	1518	1.83		
65	30.73	Cuparene	C_15_H_22_	1505	1526		0.04	
66	30.79	β-Bisabolene	C_15_H_24_	1506	1500	0.31		
67	31.12	Valencen	C_15_H_24_	1513	1504			0.29
68	31.52	Cadina-1(10),4-diene	C_15_H_24_	1523	1526			0.17
69	31.52	Methyl dodecanoate	C_13_H_26_O_2_	1523	1535	0.31		
70	31.56	β-Sesquiphellandrene	C_15_H_24_	1523	1525		2.74	
71	31.78	2-penten-1-ol	C_15_H_24_O	1528	1504		3.14	
72	32.37	trans-α-Bisabolene	C_15_H_24_	1542	1540	24.41		
73	32.55	trans-Farnesene epoxide	C_15_H_24_O	1546	1540		0.65	
74	32.76	(Z)-α-Bisabolene epoxide	C_15_H_24_O	1551	1531	1.00		
75	32.89	6,10-Dimethyl-3-(1-methylethylidene)cyclodecene	C_15_H_26_	1553	1564		0.19	
76	32.93	γ-Costol	C_15_H_24_O	1554	1542			0.94
77	33.04	Sesquirosefuran	C_15_H_22_O	1557	1577		0.15	
78	33.09	Ledol	C_15_H_26_O	1558	1566	0.16		
79	33.26	6-epi-shyobunol	C_15_H_26_O	1562	1555		0.09	
80	33.46	Globulol	C_15_H_26_O	1566	1569	0.22		
**81**	**33.52**	**1,5-Epoxysalvial-4-ene**	**C_15_H_24_O**	**1568**	**1560**		**1.07**	**0.99**
**82**	**33.98**	**Spathulenol**	**C_15_H_24_O**	**1578**	**1577**	**12.46**	**0.87**	**3.44**
**83**	**34.21**	**Caryophyllene oxide**	**C_15_H_24_O**	**1583**	**1582**	**16.88**	**7.18**	**21.89**
**84**	**34.50**	**1,2-Dihydronerolidol**	**C_15_H_28_O**	**1590**	**1574**		**0.27**	
85	34.63	6-(1-methylethyl)-1-naphthalenol	C_15_H_24_O	1593	1584			1.51
86	34.64	(−)-Globulol	C_15_H_26_O	1593	1590	0.51		
**87**	**34.81**	**Mintketone**	**C_15_H_24_O**	**1597**	**1579**		**1.00**	**1.53**
88	35.13	Rosifoliol	C_15_H_26_O	1603	1598	0.33		
89	35.15	Cedrol	C_15_H_26_O	1603	1583		8.35	
90	35.27	Copaborneol	C_15_H_26_O	1606	-			0.46
**91**	**35.45**	**Humulene epoxide**	**C_15_H_24_O**	**1609**	**1592**	**0.25**	**4.75**	**2.62**
92	35.68	Octahydro-2,2,7a-trimethyl-4-methylene-1,3a-ethano-3aH-inden-5-ol	C_15_H_24_O	1613	1599			3.33
93	35.90	Allohimachalol	C_15_H_26_O	1617	-			0.48
94	36.12	Z-3-Hexadecen-7-yne	C_16_H_28_	1621	1637			0.38
95	36.30	Aromadendrane-4,10-diol	C_15_H_26_O_2_	1624	1619			0.26
96	36.37	Agarospirol	C_15_H_26_O	1626	1608		0.89	
97	36.45	Bisabolol	C_15_H_26_O	1627	1625			0.20
**98**	**36.63**	**10,10-Dimethyl-2,6-dimethylenebicyclo[7.2.0]undecan-5-ol**	**C_15_H_24_O**	**1630**	**1647**	**0.23**		**0.67**
99	36.78	α-acorenol	C_15_H_26_O	1633	1635		1.47	
**100**	**36.81**	**11,11-Dimethyl-4,8-dimethylenebicyclo[7.2.0]undecan-3-ol**	**C_15_H_24_O**	**1633**	**1651**	**0.41**		**1.20**
101	36.92	Isospathulenol	C_15_H_24_O	1636	-	0.34		
**102**	**37.18**	**α-Cadinol**	**C_15_H_26_O**	**1640**	**1637**		**0.28**	**1.35**
103	37.40	Cedrenol	C_15_H_24_O	1644	1636			1.99
104	37.44	γ-Gurjunenepoxide-(2)	C_15_H_24_O	1645	1628		0.83	
**105**	**37.68**	**Sesquibenihiol**	**C_15_H_24_O**	**1649**	**1653**	**0.14**	**0.30**	
106	37.70	(−)-Spathulenol	C_15_H_24_O	1650	1636			5.14
107	37.88	Himbaccol	C_15_H_26_O	1653	1631	1.60		
108	37.89	(1S,4R,5S)-1-Methyl-4-(prop-1-en-2-yl)spiro[4.5]dec-7-ene-8-carbaldehyde	C_15_H_22_O	1653	1642			0.80
109	37.90	Dehydronerolidol	C_15_H_24_O	1653	1632		6.28	
**110**	**38.57**	**trans-Longipinocarveol**	**C_15_H_24_O**	**1665**	**1659**	**2.84**		**5.87**
111	38.79	Isovalencenol	C_15_H_24_O	1669	-	0.28		
112	38.87	3-Isopropyl-6,7-dimethyltricyclo[4.4.0.0(2,8)]decane-9,10-diol	C_15_H_26_O_2_	1671	1687			0.23
113	38.96	3,7,11-Trimethyl-dodeca-2,4,6,10-tetraenal	C_15_H_22_O	1672	1664		3.26	
114	39.07	6-Isopropenyl-4,8a-dimethyl-1,2,3,5,6,7,8,8a-octahydro-naphthalen-2-ol	C_15_H_24_O	1674	1690			2.02
115	39.36	1-Isopropyl-4,8-dimethylspiro[4.5]dec-8-en-7-ol	C_15_H_26_O	1680	1660		0.28	
116	39.41	Germacra-4(15),5E,10(14)-trien-1β-ol	C_15_H_24_O	1680	-			14.10
117	39.64	4-(1,5-Dimethylhex-4-enyl)cyclohex-2-enone	C_14_H_22_O	1685	1661		12.90	
118	40.53	(−)-Isolongifolol, acetate	C_17_H_28_O_2_	1701	1719			1.19
119	40.55	m-Camphorene	C_20_H_32_	1701	-		1.79	
**120**	**41.03**	**Pentadecanal**	**C_15_H_30_O**	**1710**	**1701**	**1.65**		**0.19**
121	41.05	Cuparenal	C_15_H_20_O	1710	-		3.46	
122	41.21	α-Costol	C_15_H_24_O	1713	-			0.19
123	42.23	Platambin-1,6-dione	C_15_H_22_O_2_	1731	1740			0.38
124	42.62	Epicedrol	C_15_H_26_O	1738	1743			0.81
125	43.62	Ylangenol	C_15_H_24_O	1757	-			2.33
**126**	**43.98**	**Dehydrosaussurea lactone**	**C_15_H_20_O_2_**	**1763**	**1755**		**5.49**	**4.85**
127	44.32	Valerenic acid	C_15_H_22_O_2_	1769	1759			1.53
128	45.09	β-Acoradienol	C_15_H_24_O	1783	-		2.42	
129	45.18	4a,5-Dimethyl-3-(prop-1-en-2-yl)-1,2,3,4,4a,5,6,7-octahydronaphthalen-1-ol	C_15_H_24_O	1785	-	0.58		
130	45.92	Aristol-1(10)-en-9-ol	C_15_H_24_O	1798	1788	0.93		
131	46.27	Naphthalenemethanol	C_15_H_24_O	1805	-			1.16
132	46.64	(1-Methyldecyl)cyclohexane	C_17_H_34_	1811	1790		0.35	
133	47.45	β-Copaen-4α-ol	C_15_H_24_O	1826	1820	1.96		
134	48.04	Sativene epoxide	C_15_H_24_O_3_	1837	1812		0.13	
135	48.10	(+)-Cycloisolongifol-5-ol	C_15_H_24_O	1838	-	1.80		
136	48.62	2,15-Hexadecanedione	C_16_H_30_O	1848	1864	0.66		
**137**	**48.73**	**Phytone**	**C_18_H_36_O**	**1850**	**1844**		**7.83**	**2.74**
138	48.94	6-Methyl-2-(4-methylcyclohex-3-en-1-yl)hepta-1,5-dien-4-ol	C_15_H_24_O	1854	1868	1.00		
139	49.24	15-Hydroxy-α-muurolene	C_15_H_24_O	1859	-	0.35		
**140**	**50.06**	**Diisobutyl phthalate**	**C_16_H_22_O_4_**	**1875**	**1888**		**1.09**	**0.29**
141	50.62	1-Hexadecanol	C_16_H_34_O	1885	1870			0.49
142	50.87	11-Hexadecyn-1-ol	C_16_H_30_O	1890	1872	0.44		
143	51.21	Z,Z,Z-4,6,9-Nonadecatriene	C_19_H_34_	1896	1934	0.32		
144	51.87	Geranyl-α-terpinene	C_20_H_32_	1908	1962	0.84		
**145**	**52.75**	**Farnesyl acetone**	**C_18_H_30_O**	**1924**	**1909**		**1.17**	**0.59**
146	53.16	Methyl isoheptadecanoate	C_18_H_36_O_2_	1932	1909			0.19
**147**	**54.25**	**Isophytol**	**C_20_H_40_O**	**1952**	**1939**		**0.26**	**0.36**
**148**	**55.04**	**Butyl isobutyl phthalate**	**C_16_H_22_O_4_**	**1967**	**1973**		**0.99**	**0.49**
**Sum**						**98.72**	**93.89**	**91.35**

*RI*: Experimental retention indices relative to C_8_-C_20_*n*-alkanes; *RL*: Literature retention indices. Compounds shared in common in two or three essential oils are in bold.

## Supplementary Materials

The following are available online at http://www.mdpi.com/1420-3049/23/9/2185/s1, Figure S1: Effects of SO, SG and SP essential oil on cell viability of RAW264.7. Figure S2: Linear regression analysis of caryophyllene oxide peak area percentages and IC_50_ of Hep3B and Hela cytotoxicity. Figure S3: Multiple alignment of the ITS1-5.8S-ITS2 sequence of SO, SG and SP.
