# Supplementary material for: Comparative Analysis of Chemical Composition, Anti-Inflammatory Activity and Antitumor Activity in Essential Oils from Siegesbeckia orientalis, S. glabrescens and S. pubescens with an ITS Sequence Analysis
Source: Molecules. 2018 Aug 30;23(9):2185. doi: 10.3390/molecules23092185 (PMC6225276; doi:10.3390/molecules23092185)
Supplement: Supplementary file 1 [file molecules-23-02185-s001.pdf]

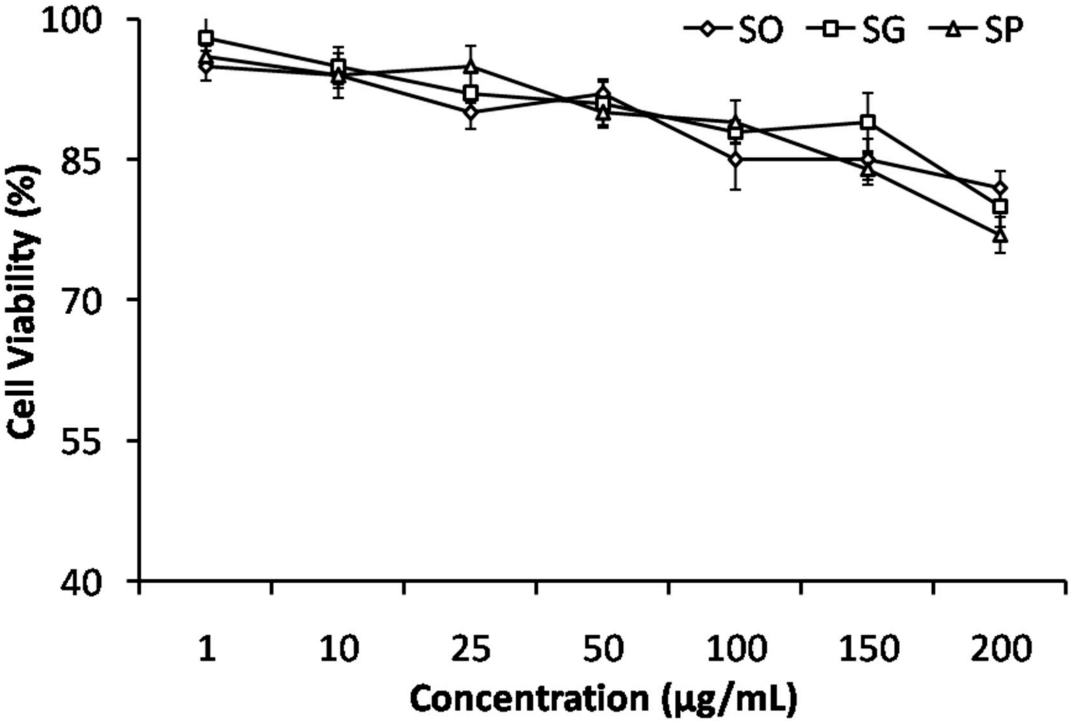

Fig. S1 Effects of SO, SG and SP essential oil on cell viability of RAW264.7

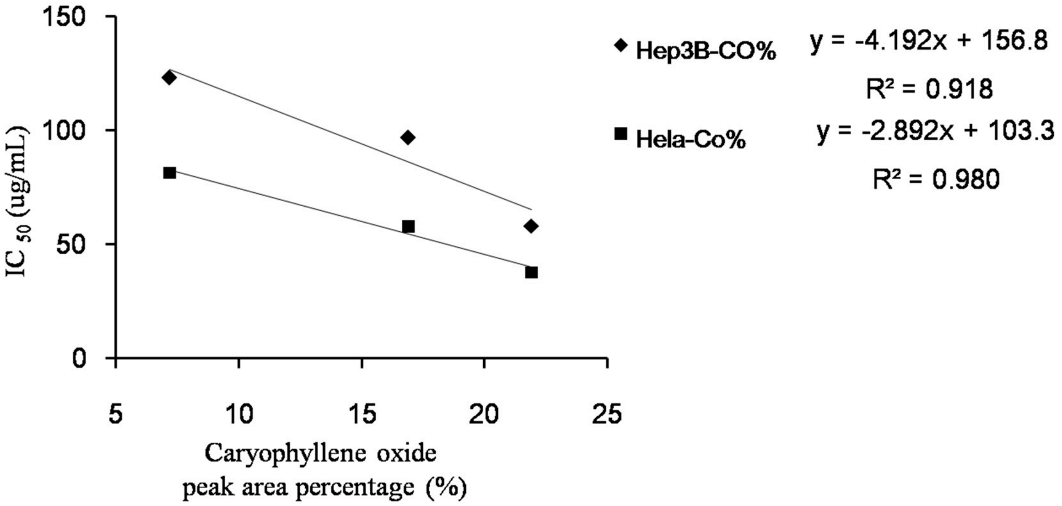

Fig. S2 Linear regression analysis of caryophyllene oxide peak area percentages and IC_{50} of Hep3B and Hela cytotoxicity.

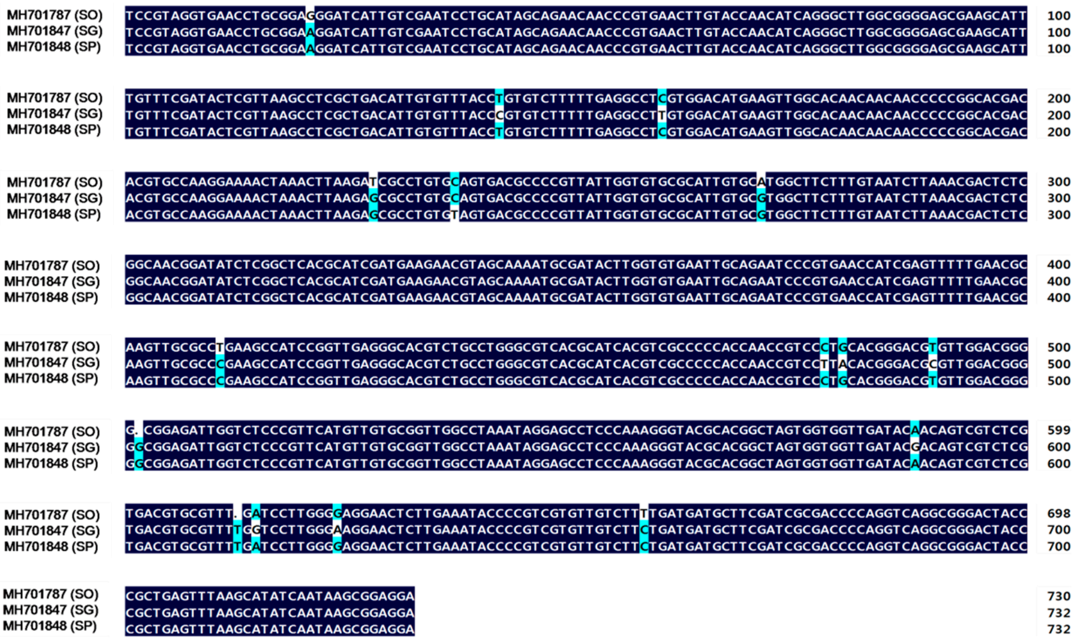

Fig. S3 Multiple alignment of the ITS1-5.8S-ITS2 sequence of SO, SG and SP
